# Molecular Mechanisms of Depression: The Interplay Between Genes and Receptors

**DOI:** 10.3390/ijms262311325

**Published:** 2025-11-23

**Authors:** Anamaria Oatu, Simona Trifu, Elena Coman

**Affiliations:** 1 Department of Psychiatry, “Iuliu Hatieganu” University of Medicine and Pharmacy, 400012 Cluj-Napoca, Romania; oatu_anamaria@elearn.umfcluj.ro; 2 Department of Neurosciences, “Carol Davila” University of Medicine and Pharmacy, 020021 Bucharest, Romania; 3 Doctoral School, “Carol Davila” University of Medicine and Pharmacy, 020021 Bucharest, Romania; elena.coman82@gmail.com

**Keywords:** depression, neuroplasticity, receptor, gene

## Abstract

Major depressive disorder arises from complex interactions between genetic variation, environmental influences, and receptor-mediated signaling that regulate mood, cognition, and stress resilience. This review synthesizes recent empirical evidence examining how genetic and epigenetic variation intersect with receptor function, binding, and expression to shape depressive phenotypes and treatment outcomes. Findings are organized into ten interconnected biochemical domains: monoaminergic, glutamatergic, GABAergic, neuropeptidergic, hormonal-metabolic, immuno-inflammatory, neurotrophic-plasticity, epigenetic/gene–environment, opioidergic and emerging therapeutics, with summary tables included for most domains to aid cross-system interpretation. Across these pathways, convergent receptor–gene relationships highlight integrative themes such as multi-omics approaches, in vivo receptor imaging, single-cell resolution mapping, and circuit-level analyses. Collectively, these findings position receptor systems as central hubs linking genetic risk and environmental modulation, providing a translational framework for receptor-centric, precision-psychiatry interventions.

## 1. Introduction

Mood disorders are among the most commonly diagnosed severe psychiatric conditions, with major depressive episodes presenting in both unipolar and bipolar forms of depression. In unipolar depression, individuals experience alternating periods of normal mood (euthymia) and depressive states, whereas bipolar disorder encompasses not only depressive episodes but also pathological mood elevations, such as hypomania or mania. According to the Diagnostic and Statistical Manual of Mental Disorders, Fifth Edition (DSM-5), a major depressive episode is defined by a cluster of symptoms, including significant disturbances in sleep, appetite, psychomotor activity, cognitive function, and mood regulation [1].

In the United States alone, major depressive disorder (MDD) affects approximately 26,1% of women and 14,7% of men throughout their lifetimes [2]. Beyond its prevalence, depression has emerged as a primary cause of suicide, accounting for nearly 44,000 deaths each year and ranking among the top ten leading causes of mortality [3]. Additionally, depression contributes to a noticeable reduction in life expectancy, not only through suicide but also due to increased vulnerability to various medical conditions such as cardiovascular disease, stroke, autoimmune disorders, diabetes, and certain cancers [4,5]. Patients suffering from depression often face heightened risks for these comorbidities and exhibit poorer prognoses when managing such illnesses [6,7]. On a global scale, major depression has become the foremost cause of disability and imposes a heavy economic burden through reduced productivity and increased healthcare costs [8].

Several factors are recognized as contributing to the onset and persistence of depression. Twin studies and research on adopted individuals suggest that roughly 40% of depression risk is genetically determined [9]. Over the past decade, research has transitioned from single candidate genes toward systems-level approaches that interrogate entire receptor networks, epigenetic modifications, and their modulation by environmental stressors [10]. This review provides an updated descriptive narrative of 203 original investigations published mainly between 2015 and 2025, focusing on how genetic variation and receptor dynamics jointly contribute to depressive phenotypes and treatment response.

Contemporary treatment strategies for depression encompass three principal approaches: (1) pharmacotherapy involving antidepressants and adjunctive medications [11], (2) psychotherapeutic interventions such as cognitive-behavioral therapy (CBT) and interpersonal psychotherapy (IPT) [12], and (3) non-pharmacological somatic therapies, including electroconvulsive therapy (ECT) [13], repetitive transcranial magnetic stimulation (rTMS) [14], and vagus nerve stimulation (VNS) [15]. Without effective treatment, patients frequently experience recurrent depressive episodes, which tend to increase in both frequency and severity over time [16].

Emerging evidence across genetics, epigenetics, receptor biology, neuroimaging, and translational studies suggests that depression cannot be explained by a single pathway. Instead, multiple receptor systems interact as interconnected networks that shape vulnerability, symptom profiles, and treatment responses. This review therefore proposes a multidimensional receptor network model of depression, highlighting how integrated multi-omics approaches, longitudinal clinical phenotyping, and receptor-targeted therapies can inform individualized treatment strategies.

## 2. Materials and Methods

We conducted research on PubMed on 15 January 2025 using the terms “Genes AND receptors AND depression”. We retained empirical studies (human or animal) that examined: (i) genetic or epigenetic variation (SNPs, CNVs, methylation, chromatin marks); AND (ii) receptor binding, density, expression, or functional assays relevant to depression pathophysiology or therapy. We extracted receptor type, gene, sample characteristics, methodology, main result, and we tried to resolve the discrepancies and duplicates—“small sample size”, considered more or less significant, indicates which receptor system a finding belongs to when a paper spans multiple pathways. Findings were grouped into ten-chapter headings defined by the predominant ligands + receptors + downstream cascades that interact to influence mood circuitry, as Table 1 shows. Emphasis was placed on replicated associations, convergence across modalities, and translational significance.

The key points and methodological themes that emerged are:

- integrative multi-omics strategies, uniting genomic, transcriptomic, and epigenomic layers to elucidate receptor-mediated mechanisms;
- advanced imaging: advanced in vivo neuroimaging, particularly PET modalities, refining quantification of receptor dynamics across human cohorts;
- single-cell resolution: high-resolution single-cell and spatial transcriptomics approaches to map receptor expression with cellular specificity;
- circuit interventions: sophisticated circuit-level manipulations in preclinical models to probe causal pathways;
- clinical phenotyping: advanced digital and clinical phenotyping paradigms that synergize behavioral and molecular datasets, refining the temporal and mechanistic resolution of depressive phenotypes.

## 3. Results

### 3.1. Serotonin Signaling

The serotoninergic system offers a well characterized entry point for understanding receptor–gene interactions in mood regulation. Because serotonin receptors, transporters and signaling cascades have been examined across molecular, imaging and pharmacological levels, this section illustrates how multiple layers of evidence converge within depressive pathophysiology.

Since 1958, serotonin has been linked to depressive states, and in 1967, Shaw et al. in post-mortem assays showed significantly lower hind-brain 5-HT concentrations in people who had died by suicide during a depressive episode, one of the first biochemical reports to connect serotonin depletion with depressive illness [17].

Serotonin (5-HT) regulates mood and anxiety through receptors such as 5-HT1A, which functions as an autoreceptor to limit serotonin release and as a heteroreceptor to mediate postsynaptic signaling. Additional work further clarifies how this polymorphism shapes receptor regulation and clinical risk. The rs6295 polymorphism in the *HTR1A* promoter affects 5-HT1A expression via the transcription factor Deaf-1, altering serotonergic activity and contributing to anxiety and depression mechanisms [18]. High-resolution PET studies in patients with MDD have demonstrated reductions in 5-HT1A binding potential in cortical and limbic regions, areas enriched in heteroreceptors. These binding deficits have been associated with the rs6295 G allele and correlate with greater hopelessness and slower response to SSRIs. This supports a mechanistic pathway in which the rs6295 G allele alters Deaf-1-mediated transcriptional repression, leading to autoreceptor predominance, reduced cortical 5-HT1A availability, and ultimately disrupted serotonergic regulation of mood [19,20,21].

The G allele of rs6295 is associated with increased risk of depression, suicide, and substance abuse [22,23]. This polymorphism affects transcription factor binding, particularly NUDR/DEAF-1, leading to altered 5-HT1A expression in raphe neurons and cortical regions [23,24]. Elevated 5-HT1A receptor binding potential appears to predict a poorer clinical response to standard antidepressant therapies, highlighting a potential biomarker for treatment resistance [25,26]. Animal studies demonstrate that increased 5-HT1A autoreceptor levels result in stress vulnerability and reduced antidepressant efficacy [27]. PET imaging reveals genotype-dependent differences in 5-HT1A distribution in depressed patients [20,28]. Understanding the complex interplay between genetics, environment, and 5-HT1A regulation may lead to improved treatments for depression and anxiety [24].

Beyond genetic factors, pharmacological approaches targeting 5-HT1A signaling also illustrate the functional relevance of serotonergic pathways. NLX-101 (F15599) is a highly selective and efficacious biased agonist of cortical 5-HT1A receptors, exhibiting a ~25-fold Gαi:β-arrestin bias compared to 8-OH-DPAT [29,30]. This biased agonism allows for preferential activation of cortical heteroreceptors without overactivating somatodendritic autoreceptors [31]. NLX-101 demonstrates rapid and sustained antidepressant-like effects in various animal models, including the forced swim test and chronic mild stress model [32,33]. Its antidepressant action is mediated through increased glutamate and dopamine release in the prefrontal cortex, as well as activation of pmTOR, pERK1/2, and pAkt signaling pathways [33]. In a phase-IIa trial, NLX-101 showed promising antidepressant efficacy without sexual side effects or treatment-emergent dissociation [29]. These findings validate the potential of biased GPCR therapeutics for mood disorders [34,35].

Recent studies have explored the role of DNA methylation in the serotonin transporter gene (*SLC6A4*) promoter region in predicting antidepressant treatment response for MDD. Hypomethylation of specific CpG sites in the *SLC6A4* promoter has been associated with impaired response to serotonergic antidepressants [36,37]. This effect was replicated in a large, independent sample and held true for patients treated with SSRIs or SNRIs. The mechanism may involve increased gene expression and decreased serotonin availability, counteracting antidepressant effects [37]. However, one study found that increased methylation at a specific CpG site (cg05016953) was associated with poorer treatment response [38]. The 5-HTTLPR genotype was not associated with *SLC6A4* methylation or treatment response in some studies [37,38]. These findings highlight the complex interplay between genetic and epigenetic factors in regulating SERT function and antidepressant response [39].

Recent research has focused on trace amine-associated receptor 1 (TAAR1) as a promising target for treating psychiatric disorders, particularly schizophrenia. TAAR1 modulates dopaminergic, serotonergic, and glutamatergic neurotransmission [40,41]. Preclinical studies have shown that TAAR1 agonists exhibit antipsychotic, anxiolytic, and antidepressant-like effects [42]. Ulotaront (SEP-363856), a TAAR1 agonist, has demonstrated efficacy in treating schizophrenia symptoms in clinical trials [43,44]. It may also improve cognitive function and reduce compulsive reward-seeking behavior. In Table 2 we present the monoaminergic gene-recveptor findings. TAAR1 activation has been associated with antidepressant-like effects and enhanced attention [45]. Additionally, TAAR1 has been linked to sleep regulation and addiction [46]. While more research is needed, TAAR1 agonists represent a promising new class of drugs for treating various psychiatric disorders, including schizophrenia, depression, and anxiety [47].

#### 5-HTTLPR Gene–Environment Meta-Analysis: Update

Recent meta-analyses have examined the interaction between the serotonin transporter gene polymorphism (5-HTTLPR) and environmental factors in relation to mental health outcomes. While some studies found a significant interaction between 5-HTTLPR and stress in predicting depression [61], others reported inconsistent results for antisocial behavior [62].

A meta-analysis of 54 studies found strong evidence that the short (S) allele of the serotonin transporter gene (5-HTTLPR) interacts with stress to increase depression risk (*p* = 0.00002), particularly for childhood maltreatment and medical conditions [58]. This supports earlier findings of gene-environment interaction [63]. However, two meta-analyses reported no significant interaction [64,65]. The discrepancy may be due to differences in included studies and stress measures [66]. Studies using objective stress measures tend to replicate the interaction more consistently than those using self-report measures [66].

The short (S) allele of 5-HTTLPR was associated with increased risk of violent suicide attempts [67] and eating disorders in females exposed to traumatic events [68,69]. Additionally, the S allele was linked to higher risks of MDD and alcohol dependence [70]. Regarding antidepressant response, long (L) allele carriers showed better outcomes and fewer adverse reactions, particularly among individuals of European descent [71]. However, some researchers suggest that very large genomic datasets are needed to definitively settle the question of 5-HTTLPR’s role in depression [72].

Epigenetic mechanisms like *SLC6A4* methylation may mediate this interaction, potentially explaining increased vulnerability of S allele carriers to stress-induced psychopathology [73].

### 3.2. Dopamine Pathways

Dopamine pathways provide a complementary perspective on depression by linking genetic variation in receptor function with alterations in reward processing and motivational states. This section outlines key findings on how dopaminergic signaling contributes to depressive symptoms and treatment response.

Actual research has shed light on the role of dopamine receptor genes in reward processing and depression. The DRD2/ANKK1 Taq1A polymorphism (rs1800497) affects striatal D2 receptor binding, with the A1 allele associated with reduced binding in healthy individuals but increased binding in depression [74,75]. This polymorphism also impacts reversal learning and associated neural activity [76]. The DRD3 Ser9Gly polymorphism (rs6280) influences reward-related dopamine release in the striatum, with the glycine allele associated with greater release [74]. Dopaminergic enhancement can increase striatal response to rewards in depression [77]. Model-based analyses suggest that major depression primarily affects reward sensitivity rather than learning rate [78]. These findings highlight the complex interplay between genetic variations, dopamine signaling, and reward processing in depression, offering potential targets for personalized treatments.

Recent meta-analysis on Pramipexole augmentation showed a 62.5% response rate in treatment-resistant unipolar and bipolar depression [79]. In Parkinson’s disease with depression, pramipexole significantly improved depressive symptoms and motor function without increasing adverse events [80,81]. For bipolar depression, pramipexole as an add-on therapy demonstrated improved response rates [82]. Pramipexole also showed beneficial effects on mood and motivational symptoms in Parkinson’s disease patients [83]. The drug’s high affinity for D3 receptors and its neuroprotective properties provide a rationale for its use in depression [79]. However, researchers emphasize the need for further high-quality randomized controlled trials to validate these findings and explore pramipexole’s potential in treatment-resistant depression [84,85].

### 3.3. Glutamatergic Plasticity

Glutamatergic mechanisms offer a mechanistic link between stress exposure, synaptic remodeling, and rapid-acting antidepressant effects. This section highlights key molecular nodes that shape plasticity in depression and inform emerging therapeutic strategies.

Impaired synaptic plasticity, not simply excess or deficit of glutamate, lies at the core of MDD. In this framework, four molecular “control knobs” stand out: the GluN2B-containing NMDA receptor (gene *GRIN2B*), the group-I metabotropic receptor mGluR5 (gene *GRM5*), the presynaptic group-II receptors mGluR2/3, and a growing family of NMDA-receptor modulators with subunit- or site-selective actions.

Post-mortem RNA-seq of anterior cingulate cortex from depressed patients shows a ~30–40% up-regulation of *GRIN2B* and a fall in the *GRIN2A:GRIN2B* ratio, consistent with a shift toward high-calcium, plasticity-potentiating GluN2B channels. The authors argue that this may raise local excitotoxic pressure and sensitise circuits to stress [86]. Stress itself appears to tune this switch: chronic restraint in mice boosts phosphorylation of GluN2B at Tyr-1070, a modification that prolongs channel open time, and genetic disruption of this site blunts depression-like behaviour [87]. Pharmacologically, GluN2B-selective negative allosteric modulators such as radiprodil normalise the gain-of-function currents produced by pathogenic *GRIN2B* mutations and are being repositioned for mood disorders [88].

High-resolution PET now resolves mGluR5 availability in living patients. Two independent cohorts report lower cortical mGluR5 binding in unmedicated MDD, with binding in the hippocampus inversely tracking symptom severity [89].

Intriguingly, a ketamine infusion transiently further suppresses mGluR5 ligand binding, hinting that down-regulation of this receptor may be integral to the antidepressant cascade rather than merely compensatory [90]. These findings dovetail with pre-clinical work in which mGluR5 negative allosteric modulators (e.g., MTEP, GRN-529) produce robust antidepressant-like effects, presumably by curbing postsynaptic glutamate spill-over and resetting metaplastic thresholds.

Whereas mGluR5 sits postsynaptically, mGluR2/3 act as autoreceptors and gate glutamate release. A 2023 translational review synthesising a decade of rodent work shows that antagonists, rather than agonists, of mGluR2/3 consistently mimic ketamine: single doses lift forced-swim immobility, restore sucrose preference, and trigger mTOR- and BDNF-dependent spine formation, without dissociation or abuse liability [91]. This presynaptic disinhibition may amplify phasic signalling through “plasticity-receptive” GluN2B and AMPA receptors, supporting the view that rapid antidepressants work by creating a brief permissive window for synaptogenesis.

Ketamine and its S-enantiomer reset mood within hours but carry psychotomimetic baggage. Comprehensive reviews from 2022–23 map the downstream pathway, in which interneuron NMDAR (NMDA receptor) blockade leads to cortical disinhibition, which in turn triggers burst firing and mTOR-synaptogenesis, and emphasize the need for safer compounds that maintain this cascade [92,93].

Together, these findings outline that: presynaptic mGluR2/3 set the amplitude of glutamate bursts; postsynaptic mGluR5 senses cumulative synaptic use and couples to intracellular Ca^2+^ stores; GluN2B-rich NMDARs integrate high-frequency input and gate BDNF-dependent spine growth; drug modulators tweak one or more of these steps. Table 3 summarises these findings.

#### NMDA Receptor-Targeted Therapies: Basic to Clinical Continuum

Over the past decade, NMDAR pharmacology has moved from the simple idea of “block the channel” to a far richer clinical-to-basic dialogue in which sub-cellular localization, subunit composition, and circuit state determine whether the same receptor is neuroprotective, excitotoxic, or therapeutically exploitable.

The discovery that a single subanesthetic dose of ketamine produces hours to days of symptom relief in treatment-resistant depression ignited an unprecedented translational cycle. Clinical trials and meta-analyses now show robust, rapid reductions in MADRS scores and suicidal ideation with intravenous, intranasal, or subcutaneous ketamine/esketamine, while basic work has traced the downstream cascade to NMDAR blockade on GABAergic interneurons, disinhibition of pyramidal firing, mTOR-BDNF signalling and spine remodeling [93,100].

Beyond ketamine, medicinal-chemistry programs have pursued novel NMDA-targeting modulators as potential rapid-acting antidepressants, including dextromethorphan and dextromethadone, lanicemine, nitrous oxide, 4-chlorokynurenine, rapastinel, apimostinel and zelquistinel. These approaches build on ketamine’s mechanisms, NMDA antagonism on GABAergic interneurons and differential effects at synaptic versus extrasynaptic receptors across brain networks [93]. Downstream consequences include enhanced AMPA throughput, activation of mTOR and BDNF-dependent plasticity, and rapid synaptogenesis [93]. High-resolution structural work using cryo-EM and artificial-intelligence-driven ligand design (AIDD) is accelerating identification of subunit-selective binding pockets and in silico optimization for next-generation agents [101].

### 3.4. GABAergic Inhibition

GABAergic dysfunction represents the inhibitory counterpart to glutamatergic dysregulation and provides a mechanistic bridge between stress exposure, genetic vulnerability, and circuit-level imbalance in depression.

Over the past 10 years, converging evidence has placed deficient GABAergic inhibition at the heart of depressive pathophysiology. Post-mortem and imaging work shows fewer GABA-ergic interneurons and lower GAD67/parvalbumin expression in prefrontal cortex, producing a persistent excitation–inhibition (E/I) imbalance that tracks with affective symptoms [102,103]. Chemogenetic studies extend this causal link: silencing parvalbumin- or CCK-positive interneurons in the basolateral amygdala precipitates depressive- and anxiety-like behaviours, whereas their activation rescues mood, underscoring circuit specificity [104]. Epigenetic repression of GAD67 via DNMT1 in the central amygdala similarly drives pain-related depression, pointing to gene–environment interactions that erode inhibitory tone [105].

Therapeutically, multiple approaches now target this deficit. Modulators of GABA-A receptors, such as brexanolone (federally approved in 2019 for postpartum depression) and zuranolone (approved in 2023), are established treatments, whereas α5-selective positive allosteric modulators have been explored in preclinical in silico models simulating human depression microcircuits, where EEG biomarkers were used to predict optimal α5-PAM dosing [106,107]. Classical antidepressants may also indirectly restore inhibition—fluoxetine reduces Dlx5/6 in interneurons in preclinical mouse studies using a control model, normalizing network rhythms and behaviour [108]. Collectively, these findings position impaired GABAergic control as a unifying mechanism linking stress, genetic vulnerability, and endocrine factors to macro-scale circuit dysfunction in depression, while highlighting interneuron protection, epigenetic editing, and receptor-selective pharmacology as fertile directions for next-generation antidepressant development.

#### GABA-A Receptor Pharmacology: Beyond Neurosteroids

GABAergic dysregulation in MDD has two recurring signatures: a shortage of transmitter caused by repressed GABA-related genes and interneuron loss, and faulty receptor trafficking that depletes synaptic inhibition. Recent work also shows that these deficits can be pharmacologically repaired, restoring mood and cognitive function.

Recent research has highlighted the potential of GABA-A receptor modulators, particularly those targeting α5-containing receptors, in treating affective and cognitive symptoms of depression. A 2023 review catalogued subtype-selective GABA-A modulators and their therapeutic potential [109]. Studies have shown that enhancing α5-containing GABA-A receptor activity may alleviate mood and cognitive symptoms in neuropsychiatric disorders [110]. Both positive and negative modulators of α5 GABA-A receptors have demonstrated cognitive-enhancing effects in animal models [111]. Ongoing clinical trials are exploring compounds like basmisanil for cognitive impairment and darigabat for panic disorder [112]. These α5-selective modulators offer potential for developing non-sedating anxiolytics and cognitive enhancers with fewer side effects than classical benzodiazepines [113].

GABA-A receptor abundance at synapses is set by scaffolding proteins. Two stress-responsive pathways disrupt that functioning. The first one is MDGA1–Neuroligin-2 (*Nlgn2*). Chronic stress up-regulates MDGA1 in the lateral habenula (LHb). MDGA1 blocks *Nlgn2*, limits GABAergic synapse formation, and drives depressive phenotypes; deleting MDGA1 or introducing a *Nlgn2* mutant that escapes MDGA1 restores inhibition and resilience [114]. The second one is the IL-10/gephyrin cascade. Early-life stress lowers IL-10 in the LHb. Reduced IL-10 suppresses PI3K-AKT signalling, prevents gephyrin palmitoylation, and traps GABA_A receptors inside the neuron. Over-expressing IL-10 or activating its pathway reinstates receptor trafficking and normalizes mood [115].

Recognition of these lesions has produced targeted therapies. Neuroactive steroids such as zuranolone, an oral positive allosteric modulator that prefers δ-containing extrasynaptic GABA_A receptors, deliver rapid antidepressant effects within three days in phase-3 trials and are generally well tolerated [116] (Table 4). Because they amplify endogenous GABA rather than forcing inhibition, they fine-tune tone without the cognitive dulling associated with classic benzodiazepines.

Together, these findings argue that depression is not merely “low monoamines” but, in many patients, a state of cortical disinhibition. Genetic or stress-induced lesions diminish GABA supply or derail receptor delivery, while selective GABA_A potentiators and cytokine or adhesion-protein interventions can re-balance excitation–inhibition.

### 3.5. Neuropeptide Stress Axes

Neuropeptide stress pathways provide a complementary dimension to monoaminergic and glutamatergic models, capturing how early-life adversity and genetic variation converge on HPA-axis reactivity and limbic threat processing in depression.

The corticotropin-releasing-hormone receptor-1 (CRHR1) SNP rs110402 (A/G) is located in an intronic enhancer that modulates CRHR1 transcription. Two large imaging-genetics studies have shown that the A allele interacts with a history of childhood maltreatment to amplify limbic threat processing. In a sample of 150 young adults, A-carriers exposed to high early-life stress displayed significantly greater basolateral-amygdala BOLD responses to fearful faces than G/G homozygotes [119]. A replication in 308 participants demonstrated that a polygenic “HPA-risk” score—driven in part by rs110402—potentiated right-amygdala reactivity to threat cues after trauma [120]. Heightened amygdala activity and stronger amygdala to ventromedial prefrontal connectivity constitute a neural intermediate phenotype for stress-sensitized, anxiety-laden depressive presentations.

CRF_1_ antagonism: Despite clear genetic and imaging evidence that CRF signalling is overactive in susceptible individuals, CRF_1_-receptor blockade has not yet translated into robust clinical benefit. In a 6-week, randomised, placebo-controlled trial involving 242 adults with generalised anxiety disorder, the long-acting antagonist pexacerfont produced substantial baseline-cortisol suppression but failed to out-perform placebo on primary symptom measures [121]. The related compound verucerfont likewise suppressed cortisol in alcohol-dependent women without reducing stress-induced craving or affective symptoms [122]. Exploratory work in post-traumatic stress disorder suggests that lower baseline CRHR1 methylation and treatment-related demethylation track greater symptom improvement under CRF_1_ blockade, hinting that epigenetic or inflammatory stratifiers could identify responder subgroups [123].

Neuropeptide-Y buffering: The NPY_1_ receptor (*NPY1R*) is enriched in ventromedial-prefrontal and amygdala circuits that dampen fear. In rhesus macaques, higher *NPY1R* mRNA in the central amygdala predicts lower anxious temperament and reduced metabolic activity in threat-processing networks (Table 5) [124]. Human imaging-genetic work links NPY system variation to stronger vmPFC-amygdala connectivity and greater resilience, although direct evidence that *NPY1R* promoter hypomethylation confers stress resilience in humans has not yet been published [125].

Taken together, current data support a gene-by-environment pathway in which early trauma interacts with the CRHR1 rs110402 A allele to produce limbic hyperreactivity and increase the risk for mood and anxiety disorders. While first-generation CRF_1_ antagonists have demonstrated biological target engagement without broad clinical efficacy, emerging biomarker-guided approaches and complementary strategies that enhance NPY signaling may still achieve circuit-specific normalization of HPA-driven pathology [123].

### 3.6. Hormonal and Metabolic Receptors

Hormonal and metabolic receptors shape the neurobiology of major MDD by translating systemic signals-stress, sex, energy balance and circadian phase into synaptic change. Over the last decade, six receptor families have emerged as the most reproducibly altered in patients or as promising treatment targets: stress-axis receptors, sex-hormone receptors, thyroid-hormone receptors, metabolic receptors, circadian-metabolic crosstalk, and incretin and nuclear receptors.

Epigenetic studies show that childhood adversity leaves a methylation “scar” on the *NR3C1* promoter; higher methylation predicts blunted glucocorticoid-receptor (GR) transcription and more severe depressive symptoms two years later in adolescents [126]. GR hypofunction perpetuates cortisol hypersecretion, a hallmark of melancholic and metabolic subtypes of depression [127,128].

Declining ovarian estradiol unmasks mood vulnerability during the perimenopause. Contemporary reviews integrate cellular, rodent and clinical data to show that ER-α and ER-β signalling maintains synaptic plasticity in corticolimbic circuits; reduced ER tone is now linked to cognitive impairment and anhedonia in women with MDD [129].

Triiodothyronine (T3) acts via nuclear TRα/β to speed cortical metabolism. A 2018 synthesis of 353 patients reported that supraphysiological T3 consistently accelerated or augmented antidepressant response, particularly in bipolar depression, despite the small size of individual trials [130].

Central insulin resistance is increasingly viewed as a treatable driver of affective illness. In a 2022 case study of treatment-resistant bipolar depression, reversal of insulin resistance with metformin was accompanied by restoration of blood–brain barrier integrity and full symptomatic remission, implicating brain insulin-receptor signalling in mood restoration [131]. Leptin receptors add another metabolic dimension: transcriptomic work in late-onset depression identified *LEP* as a high-accuracy diagnostic marker, linking dysregulated leptin signalling to inflammatory depression phenotypes [132].

A 2023 meta-analysis (5 trials, 1 cohort study, >2000 participants) revealed that glucagon-like-peptide-1 receptor agonists (GLP-1RAs) such as liraglutide and semaglutide produced moderate reductions in depression scores versus control treatments, independent of weight loss [133]. Peroxisome-proliferator-activated-receptor-γ (PPAR-γ) agonism shows parallel promise: a double-blind trial of pioglitazone (15–45 mg) in bipolar depression improved depressive symptoms and normalised inflammatory markers relative to placebo [134].

Agomelatine, a combined melatonin MT1/MT2 agonist and 5-HT_2_C antagonist, leverages both chronobiotic and metabolic mechanisms. The data from the VIVALDI study confirm good tolerability and antidepressant efficacy for agomelatine alone or in combination therapy [135]. These findings are presented in Table 6.

### 3.7. Immuno-Inflammatory Interfaces

MDD is associated with a low-grade systemic inflammatory profile, with consistently reported increases in IL-6, CRP, and, in many studies, TNF-α, which correlates with symptom severity and treatment resistance [139,140].

Peripheral inflammation signals the brain via humoral transport, vagal afferents and effects on the blood–brain barrier, promoting microglial activation and a shift toward pro-inflammatory microglial phenotypes that impair neurogenesis and synaptic plasticity [141]. In vivo PET studies using TSPO ligands and transcriptional data support microglial upregulation in subgroups of patients with MDD [142].

Inflammation also perturbs neurotransmitter metabolism (e.g., IDO-mediated tryptophan to kynurenine flux), favouring neurotoxic kynurenine metabolites and glutamatergic excitotoxicity; moreover, anti-inflammatory interventions (e.g., TNF antagonists) have shown benefit primarily in patients with elevated baseline inflammatory markers, supporting an inflammation-targeted precision-medicine approach [143,144].

#### Microbiome-Derived Inflammatory Ligands

Depressed patients consistently display a low-grade, systemic inflammatory signature with elevated IL-6, TNF-α, and CRP, alongside a reduction of anti-inflammatory short-chain fatty acids (SCFAs) such as butyrate and propionate in plasma [145]. These humoral changes correlate with an increase in gut permeability (“leaky gut”), allowing microbiome-derived ligands, including lipopolysaccharide (LPS), peptidoglycan, and flagellin, to activate pattern-recognition receptors (PRRs) on circulating monocytes and brain-resident microglia [146].

Once inside the brain, danger signals tilt microglia toward a pro-inflammatory M1 state and prime the NLRP3 inflammasome, a molecular platform that converts pro-IL-1β and pro-IL-18 into their mature, mood-perturbing forms. Contemporary reviews highlight ATP–P2X7 receptor signaling as a crucial “second hit” that drives NLRP3 activation in rodent stress models and in post-mortem human tissue [147,148]. Pharmacological or genetic blockade of either P2X7 or NLRP3 reverses anhedonia and behavioural despair in rodents, positioning the inflammasome as a translational target for cytokine-high subgroups of MDD [149].

The microbiome supplies not only pro but also counter-inflammatory metabolites. SCFAs cross the blood–brain barrier, inhibit histone-deacetylases and bolster microglial “resting” phenotypes; exogenous SCFA administration rescues neurogenesis, repairs blood-brain-barrier integrity, and abolishes depressive-like behaviour in fructose-fed mice [150]. A second metabolite axis involves tryptophan catabolism. Gut bacteria transform dietary tryptophan into indoles and kynurenine, both of which modulate aryl-hydrocarbon and NMDARs, respectively. Dysbiosis skews this metabolic flux toward the neurotoxic quinolinic-acid arm, fueling glutamatergic excitotoxicity and depressive symptoms [151].

These mechanistic insights have spurred microbiome-directed interventions. A mechanistic RCT showed that a four-week, multi-strain probiotic not only reduced Hamilton Depression Rating Scale scores but also dampened plasma IL-6 and normalised functional connectivity within reward networks (Table 7) [152]. A subsequent JAMA Psychiatry trial (n = 49) confirmed the adjunctive efficacy and excellent tolerability of probiotics when added to SSRIs, with effect sizes comparable to those of established augmentation agents and no serious adverse events [153]. Meta-analyses now place psychobiotics alongside anti-cytokine biologics (e.g., infliximab) and small-molecule NLRP3 inhibitors as emerging options for inflammation-biased depression.

In summary, immuno-inflammatory interfaces such as cytokine signalling, PRR activation and the NLRP3 inflammasome intersect with microbiome-derived ligands and metabolites to create a self-reinforcing loop that sustains depressive pathology. Targeting this loop with dietary SCFAs, probiotic “psychobiotics,” or inflammasome modulators offers a precision-medicine route to treating the roughly one-third of patients whose mood disorder is driven by an inflammatory–microbial axis.

### 3.8. Neurotrophic and Synaptic Genes

Neurotrophic and synaptic signaling pathways provide a mechanistic bridge between molecular vulnerability and circuit-level dysfunction, highlighting how impaired trophic support and altered synapse maintenance contribute to the structural and functional deficits observed in depression.

Several large-scale genetic and transcriptomic studies implicate BDNF as a core susceptibility gene in MDD, with polymorphisms such as Val66Met (rs6265) associated with depression severity and treatment outcomes [157,158]. Gene-based analyses also highlight VEGFA and other neurotrophic pathway genes as modulators of antidepressant response in depressed cohorts [159].

At the transcriptome level, meta-analytic integration of post-mortem human brains reveals downregulation of genes encoding excitatory synaptic scaffolding proteins (e.g., *DLG4*/PSD95, *SHANK3*, *GRIA2*) in limbic regions in MDD [160].

Epigenetic regulation plays a modulatory role: hypermethylation within promoters of neurotrophic genes (particularly the BDNF exon IV locus) has been associated with reduced expression and worse antidepressant response [161,162].

These convergent genetic, transcriptomic, and epigenetic findings suggest a vulnerability in the genomic architecture that governs trophic support and synapse maintenance, which may predispose to the functional deficits seen in depression.

#### Neurotrophic and Synaptic Signaling

Meta-omic studies show reduced brain-derived neurotrophic factor (BDNF) mRNA and protein in limbic cortices of unmedicated MDD cases, with levels normalising after successful treatment [163]. Epigenetic profiling adds a mechanistic layer: hyper-methylation at the BDNF −87 promoter predicts poor antidepressant response, whereas demethylation accompanies remission, suggesting a reversible “neuroplastic brake” on the gene [164]. On the receptor side, imaging-genetic work indicates that common *NTRK2* (TrkB) variants modulate hippocampal circuitry and treatment resistance, implicating receptor signalling integrity in therapeutic outcome [165].

Post-mortem single-nucleus RNA-seq in >450 brains has revealed a concerted down-shift of genes that build and maintain excitatory synapses, PSD95 (*DLG4*), *SHANK3*, *GRIA2*, and an up-shift of microglial phagocytic genes in both subgenual cingulate and amygdala, pointing to activity-dependent synapse pruning as a canonical lesion in MDD [166]. Functionally, these transcriptional scars converge on the mTORC1 pathway that couples trophic input to dendritic protein synthesis. Pharmacological activation of mTORC1 by ketamine or its metabolite (2-R,6-R)-HNK rapidly restores spine density and mood in rodents and humans, whereas rapamycin blocks both spine gain and the antidepressant effect [167,168].

Vascular endothelial growth factor (VEGF) has emerged as a parallel trophic signal. Two recent clinical studies found that baseline plasma VEGF is elevated in a subset of treatment-resistant patients and selectively predicts response to electro-convulsive therapy or ketamine infusions [169]. VEGF crosses the blood–brain barrier, promotes hippocampal neurogenesis and acts synergistically with BDNF, making it a plausible biomarker for “growth-factor-responsive” depression [170].

High-throughput CRISPR screens and mouse models are uncovering new plasticity genes with translational traction. Over-expression of NEGR1, a synapse-adhesion gene highlighted by psychiatric GWAS, induces depression-like behaviour and impairs myelination; knocking it down rescues stress-induced anhedonia, revealing a fresh therapeutic target for synaptic repair [171].

In Table 8, we present the neurotrophic and synaptic gene findings implicated in MDD.

### 3.9. Epigenome and Gene–Environment Interplay

The epigenome acts as a dynamic interface through which stress, trauma, and environmental exposures shape gene expression across HPA-axis and neuroplasticity pathways. This framework helps explain why similar genetic backgrounds can yield different depression trajectories under different environmental loads.

*FKBP5* encodes FKBP51, a co-chaperone that associates with the cytoplasmic glucocorticoid-receptor (GR) complex. When FKBP51 expression is high, it competes with the activating co-chaperone FKBP52, thereby reducing GR ligand-binding affinity and slowing GR nuclear translocation; the net result is a higher cortisol concentration and a prolonged hormonal stress response [176].

A pivotal study showed that the T (“risk”) allele of rs1360780, located in an intronic enhancer, sensitises that enhancer to glucocorticoid signalling. In childhood-trauma survivors who carry the risk allele, repeated GR activation produces allele-specific de-methylation (not hypermethylation) at glucocorticoid-response elements in intron 7, leading to enduring *FKBP5* over-expression and exaggerated cortisol output [177].

Subsequent imaging-genetics work confirmed that rs1360780 T-carriers with lower *FKBP5* methylation display reduced cortical thickness and altered limbic connectivity—structural correlates of stress sensitization [178]. Mediation analyses across cohorts find only modest or nonsignificant mediation of the childhood-trauma → depression link by *FKBP5* methylation [179]. Thus, *FKBP5* methylation is better viewed as a dynamic marker of HPA-axis plasticity than a strong mediator of genetic risk.

Because methylation shifts at the intron-7 enhancer can be detected in peripheral blood and saliva, several studies propose *FKBP5* CpG panels as biomarkers of stress-load and GR resistance; however, clinical utility awaits replication in larger, longitudinal samples [180].

Ambient air pollution acts synergistically with polygenic vulnerability to raise future depression risk; this gene-environment interplay has been reproduced in very large cohorts but still lacks mechanistic detail [181]. Table 9 presents the air-pollution interactions with genetic risk.

Prospective work in Chinese adolescents shows that childhood maltreatment progressively increases DNA-methylation at the *NR3C1* promoter, and those methylation gains partially mediate the path from trauma to later depressive symptoms [126]. A complementary mechanism operates at the stress-responsive *FKBP5* locus: in carriers of the rs1360780 T allele, early adversity drives demethylation of intron-7 CpGs, yielding sustained FKBP51 overexpression and an exaggerated cortisol profile [180]. Thus, identical environmental input can push glucocorticoid-signalling genes in opposite epigenetic directions, depending on enhancer architecture.

Neurotrophic genes are equally malleable. Reviews integrating human and rodent data document that hypermethylation of the BDNF exon-IV promoter accompanies low serum BDNF in unmedicated MDD and normalises with successful therapy [183]. At the chromatin level, reduced histone-acetylation in prefrontal cortex, partly via cytoplasmic HDAC6, impairs synaptic protein translation; selective HDAC6 inhibition reverses behavioural despair in rodents, linking histone marks to mood control [184].

Noncoding RNAs add a third regulatory tier. Knock-down of micro-RNA-124 alleviates anhedonia and restores hippocampal CREB-BDNF signalling in chronic-stress rats, underscoring miR-regulated plasticity [185]. A 2024 report identifies a circulating circFKBP8 peptide that blocks GR nuclear translocation; its whole-blood level discriminates patients from controls and tracks symptom severity, providing a novel epigenetic biomarker anchored in stress biology [186].

Genome-wide approaches reveal that epigenetics does not act in isolation. A UK-Biobank-sized “genome-by-trauma” study estimated that common-variant interactions with childhood adversity account for up to 20% of MDD liability, fivefold higher in men than women [187]. Mendelian-randomisation analyses further show bidirectional causality between maltreatment and depression, hinting at active gene–environment correlation where genetic risk elevates exposure to adversity [188,189]. These findings dovetail with multi-omic HPA-axis studies showing that *NR3C1* and *FKBP5* methylation respond dynamically to psychotherapy, exercise and diet, suggesting that epigenetic “scars” remain pharmacologically and behaviourally reversible [190].

#### Epigenetic Signatures of Early-Life Stress

Early-life stress (ELS) induces long-lasting epigenetic modifications—including DNA methylation, histone modifications, and noncoding RNA regulation—that modulate gene expression without altering DNA sequence [191,192]. These modifications often target genes involved in stress response and neuroplasticity. Increased methylation of the *NR3C1* promoter has been associated with heightened vulnerability to depression following childhood maltreatment [193]. Similarly, *FKBP5* gene methylation alterations are observed in individuals exposed to early-life adversity, affecting glucocorticoid receptor sensitivity and HPA-axis regulation [194]. These epigenetic changes can lead to altered cortisol dynamics, increasing susceptibility to depressive symptoms [195]. Moreover, maternal prenatal depression has been linked to elevated *NR3C1* methylation in infants, suggesting a mechanism for intergenerational transmission of stress effects [196]. Collectively, these findings highlight the critical role of ELS-induced epigenetic modifications in shaping long-term vulnerability to depression.

### 3.10. Translational and Therapeutic Horizons

A decade of mechanistic drug development has pushed antidepressant discovery beyond the monoamine world and toward ligand-gated ion channels, neurosteroid sites, stress peptidergic systems, and precision muscarinic circuitry.

Esketamine, the first NMDA-receptor modulator to reach the clinic, validated glutamatergic disinhibition as a therapeutic lever: a single intranasal dose can reduce suicidal ideation and produce a ≥50% MADRS drop within 24 h, an effect sustained by twice-weekly top-ups. The drug’s rapidity has reset expectations for onset speed in severe depression [197,198].

Acting at the opposite pole of excitation–inhibition balance, the oral neurosteroid zuranolone boosts extrasynaptic GABA-A tone. In a phase-3 randomized controlled trial, a two-week course of zuranolone 50 mg once daily was sufficient to induce and maintain symptomatic relief for at least six weeks, simplifying long-term management and expanding the neurosteroid class beyond intravenous brexanolone [199].

The kappa-opioid blocker aticaprant offered anhedonia-targeted benefits in phase 2, aligning with preclinical data that KOR antagonism rescues stress-induced reward deficits. Yet the termination of its confirmatory trial warns that target engagement does not guarantee durability, underscoring the importance of translational biomarkers for patient selection [200].

Cholinergic modulation is reemerging through the M4-selective PAM emraclidine. Unlike earlier non-selective agonists, emraclidine spares cognition and gastrointestinal function by avoiding M1/M2 activation. Early imaging shows it normalises fronto-striatal signalling; depression trials will test whether M4-driven rebalancing of cortical output can lift mood without sedation [201,202].

Finally, an approximately one-million-participant GWAS has catalogued 354 depression-risk loci, heavily skewed toward synaptic and neurodevelopmental genes. These hits nominate fresh receptor classes, such as presynaptic vesicle proteins and cell-adhesion GPCRs, for medicinal-chemistry exploration, ensuring the next wave of receptor-targeted antidepressants is grounded in human genetics [203].

Together, these advances outline a nimble discovery pipeline where rapid-acting, circuit-specific agents are progressing from molecule to phase-3 trials within half a decade, carrying the promise of more personalized and mechanistically informed care for MDD.

In Table 10, we present the receptor-targeted antidepressants and genomic leads as they are seen in the last 10 years (2015–2025).

### 3.11. Rapid-Acting Antidepressants and Glutamatergic Neurobiology

Modulating glutamatergic neurotransmission has become a primary focus in the development of rapid-acting antidepressants (RAADs). Unlike monoaminergic antidepressants—whose therapeutic delay has classically been attributed to downstream neuroadaptive changes such as gradual increases in BDNF expression, synaptogenesis, and circuit remodelling [204]—Casarotto et al. [205] showed that a key rate-limiting step may instead be the time required for these drugs to accumulate sufficiently to bind the low-affinity site on the TRKB transmembrane domain, thereby enabling efficient BDNF-dependent plasticity. In their model, both typical and rapid-acting antidepressants directly bind TRKB, increase its surface localisation, and potentiate BDNF signalling.

A single sub-anaesthetic ketamine infusion produces a rapid cortical glutamate surge and increases AMPA throughput [206], but Casarotto et al. demonstrated that ketamine’s rapid antidepressant action also reflects its direct binding to TRKB, which accelerates BDNF-evoked TRKB phosphorylation and plasticity. Although ketamine can activate mTORC1-dependent structural plasticity [207], the TRKB-binding mechanism provides an additional unifying explanation for its speed of action. The intranasal S-enantiomer esketamine translates these rapid plasticity effects clinically: the ASPIRE phase-3 trials showed clinically meaningful reductions in depressive symptoms and suicidal ideation within 24 h of the first dose, maintained with twice-weekly administration [208].

The ketamine metabolite (2R,6R)-hydroxynorketamine reproduces rapid synaptogenesis without dissociative side effects [209], and in Casarotto et al., it was shown to bind TRKB as well, supporting the view that TRKB engagement is central to both ketamine and HNK effects. In parallel, NV-5138, a small-molecule sestrin-2 modulator that directly activates mTORC1 in the medial prefrontal cortex, reverses anhedonia for up to seven days after a single oral dose in rodents—an effect blocked by BDNF neutralisation [210]—further underscoring the centrality of BDNF-dependent plasticity pathways in rapid antidepressant responses.

Rapastinel, a glycine-site NMDAR modulator, enhances rather than inhibits NMDAR currents yet still produces ketamine-like, 24-h mood improvements with negligible psychotomimetic effects in early trials [211]. Dextromethorphan–bupropion (AXS-05) combines low-affinity NMDA antagonism with sigma-1 agonism; phase-3 data show significant MADRS separation from placebo after one week while maintaining good tolerability [212].

Across these molecules, glutamate burst, AMPA facilitation, and BDNF-mTORC1-dependent synaptogenesis emerge as the final common pathway. This cascade restores excitatory/inhibitory balance in medial prefrontal and hippocampal circuits that govern mood regulation.

Esketamine is now the standard of care for imminent suicide risk, while AXS-05 has secured FDA approval for major depression. Rapastinel and NV-5138 illustrate second-generation strategies that preserve speed but aim for better safety or oral convenience. Together, they confirm that targeting glutamatergic plasticity can compress antidepressant onset from weeks to hours, opening a new therapeutic era for patients who cannot wait.

### 3.12. Opioidergic Modulation

Opioid receptors, traditionally studied for their roles in analgesia and addiction, constitute a neuropeptide-based signalling axis that interfaces tightly with the circuits governing stress and mood, and thus belong logically within the “neuropeptide stress” domain of depression neurobiology [213]. Preclinical and clinical evidence indicates that μ-opioid receptor (MOR) activation has antidepressant and anxiolytic effects. Buprenorphine, a partial MOR agonist and κ-opioid receptor antagonist, produces antidepressant-like responses and reduces suicidal ideation in depressed patients, supporting MOR–KOR modulation as a potential therapeutic strategy [214].

The kappa opioid receptor (KOR) system, activated by stress-induced dynorphins, modulates mood-related neurochemistry by reducing dopamine, norepinephrine, and serotonin, contributing to depressive-like behaviors [215]. Adjunctive treatment with aticaprant significantly reduced depressive symptoms in patients with MDD who were partially unresponsive to SSRI/SNRI antidepressants, with higher response rates observed, particularly in those with elevated anhedonia. By blocking kappa receptor signaling, aticaprant may restore dopamine and serotonin release, enhancing reward processing and supporting antidepressant effects [200].

Delta (DOP) opioid receptors help regulate mood and stress responses, and their activation produces antidepressant-like effects, though some agonists may carry seizure risk [216]. These receptors play a key role in mood regulation and emotional processing. Altered DOR signaling, including reduced receptor expression in regions like the amygdala and hippocampus, has been linked to depression, anxiety, and cognitive-emotional deficits, while DOR agonists in animal models produce antidepressant-like effects and modulate stress responses [217].

Opioid receptors MOR, KOR, and DOR play important roles beyond pain modulation, directly influencing mood, stress responses, and reward processing. Targeting these receptors, through MOR agonists, KOR antagonists like aticaprant, or DOR modulators, offers a promising approach for novel antidepressant therapies, especially in patients with treatment-resistant depression or marked anhedonia (Table 11).

### 3.13. Integrative Models and Network Analyses

In recent years, the integration of multiomics approaches has significantly advanced our understanding of major MDD. By combining data from genomics, transcriptomics, proteomics, metabolomics, and other omics layers, researchers have been able to construct more comprehensive models of depression that account for its complex biological underpinnings. For instance, studies have demonstrated that combining proteomic and metabolomic data can elucidate interactions that are crucial for understanding mental health disorders [218]. Network medicine has emerged as a powerful framework for analyzing these multiomics datasets. By constructing disease-specific networks, researchers can identify key molecular players and pathways involved in depression. For example, one study utilized a network medicine approach to explore the relationship between depression and inflammation, identifying critical targets and potential therapeutic interventions [219]. These integrative models not only enhance our understanding of the pathophysiology of MDD but also hold promise for improving prediction, diagnosis, and treatment strategies. As the field progresses, the continued integration of multiomics data with advanced computational techniques will be essential for unraveling the complexities of depression and developing more effective personalized therapies.

## 4. Discussion

Recent findings demonstrate that mood disorders such as MDD reflect convergent dysfunction across multiple receptor-signaling systems rather than isolated gene effects. Several lines of evidence illustrate how specific gene variants interact with environmental and molecular factors to influence depression-related phenotypes. For example, the functional promoter variant *HTR1A* rs6295 not only alters 5-HT1A autoreceptor expression but also interacts with stress exposure to influence threat-processing and suicidality in mood disorders [22,220]. Similarly, the DRD2/ANKK1 Taq1A (rs1800497) variant modulates striatal D2/3 receptor binding in depressed individuals, pointing to dopaminergic impairments underlying anhedonia and reward blunting [55,74,221]. This polymorphism has also been linked to failure in smoking cessation and may predispose individuals to depression and addiction via its effect on the post-synaptic D2 receptor [74]. Beyond monoamines, innate immune signatures, for instance, altered TLR4 expression and methylation, emerge as key nodes linking peripheral inflammation to depressive symptom severity and treatment non-response.

The CRHR1 rs110402 G allele has been linked to increased risk for depressive symptoms following early-life stress, suggesting a gene-environment interaction that modulates stress sensitivity [222]. However, a meta-analysis found no association between CRHR1 rs110402 and depression, indicating variability in genetic risk across populations [223].

Hypermethylation of the *NR3C1* gene has been associated with altered cortisol reactivity and may serve as biomarkers for perinatal transmission of depressive risk [224]. Increased methylation of *NR3C1* and BDNF genes is associated with increased risk of depression, suggesting that DNA methylation may play a role in the pathophysiology of the disorder [225].

The BDNF Val66Met polymorphism influences hippocampal function and stress response, with Met allele carriers exhibiting attenuated rapid antidepressant and antisuicidal responses to ketamine, highlighting the importance of BDNF in synaptic plasticity and treatment outcomes [226]. Serum BDNF concentrations were significantly lower in patients with attempted suicide/ideation, suggesting that BDNF concentrations could serve as a response marker for antidepressant treatment in MDD [227].

Together with the receptor–variant interactions detailed in Table 12, these findings reinforce the concept of depression as a network disorder shaped by multilevel receptor–gene dynamics.

## 5. Conclusions

Our systematic synthesis of 203 empirical studies indicates that depression arises not from a single neurotransmitter or gene, but from a complex, interactive network of receptor-mediated processes spanning monoaminergic, glutamatergic, GABAergic, neuropeptidergic, hormonal, metabolic, immuno inflammatory, and neurotrophic systems. Across these domains, convergent evidence shows that disturbances in receptor signaling, whether caused by genetic variation, epigenetic modulation, or altered receptor expression, shape the molecular framework underlying mood dysregulation.

Integrative multi-omics research demonstrates that receptor function is dynamically influenced by interactions across genomic, transcriptomic, and epigenomic layers, while advanced in vivo imaging enables precise quantification of receptor dynamics in human populations. Single-cell and spatial transcriptomic analyses further reveal cell type and region-specific patterns of receptor expression, offering insight into interindividual differences in vulnerability and treatment response.

Experimental manipulations at the circuit level provide causal evidence for the involvement of receptor-specific pathways in mood regulation, and translational studies highlight how molecular findings inform the development of novel therapies, including NMDA and GABA-A modulators, glucocorticoid receptor regulators, and immune-targeted antidepressants.

Overall, the accumulated evidence supports a shift from monoamine-centered hypotheses toward a multidimensional receptor network model of depression, in which diverse signaling systems converge on shared mechanisms of synaptic plasticity, stress adaptation, and neuroimmune balance. Future work integrating longitudinal clinical phenotyping, comprehensive multi-omics profiling, and receptor-based therapeutic approaches will be essential to progress from molecular association to causal understanding and individualized treatment strategies.

## Figures and Tables

**Table 1:** Broad biochemical/physiological systems we used to organize the 203 findings—a functionally coherent system centered on a ligand-receptor pair (e.g., serotonin → 5-HT1A, glutamate → NMDA, or cytokine → TLR4) together with its immediate downstream transduction organ system. All findings are organized into ten such domains—monoaminergic, glutamatergic, GABAergic, neuropeptidergic, hormonal-metabolic, immuno-inflammatory, neurotrophic-plasticity, epigenetic/gene–environment, and emerging therapeutics—to condense thousands of discrete molecular observations into clinically interpretable themes.

	Section Heading	What It Covers	Representative Genes/Receptors Discussed
1	Monoaminergic systems—split into *Serotonin* (Section 3.1) and *Dopamine* (Section 3.2)	Classical neurotransmitters that mediate fast synaptic transmission and neuromodulation	*HTR1A*, *HTR2A*, *SLC6A4*, DRD2, DRD3, COMT, TAAR1
2	Glutamatergic signalling (Section 3.3)	Excitatory aminoacid transmission and its role in synaptic plasticity and rapid-acting antidepressants	*GRIN2B*, *GRM5*, mGluR2/3, NMDA-receptor modulators (ketamine, rapastinel)
3	GABAergic inhibition (Section 3.4)	The brain’s chief inhibitory system and its extrasynaptic “tonic” control of network excitability	*GAD1* (GAD67), SST interneuro, *MDGA1*/*Nlgn2*, GABA_A receptor–gephyrin, GABA_A δ subunit
4	Neuropeptide stress axes (Section 3.5)	Peptides that orchestrate the stress response and resilience	CRHR1, *NPY1R*
5	Hormonal & metabolic receptors (Section 3.6)	Endocrine and metabolic messengers that couple body physiology to mood	*NR3C1*, ER-α/β, TR-α/β, Central insulin receptor signalling, GLP-1 receptor, PPAR-γ receptor, MT1/MT2
6	Immuno-inflammatory interfaces (Section 3.7)	Cytokine, toll-like and purinergic signalling that link peripheral/central inflammation to depression	IL-6, TLR4, *P2RX7*, FFAR2 (GPR43), IDO1
7	Neurotrophic & synaptic-plasticity genes (Section 3.8)	Growth-factor pathways that remodel synapses and circuits	BDNF, *NTRK2* (TrkB), Synaptic scaffolds (PSD95/*DLG4*, *SHANK3*, *GRIA2*), mTORC1 pathway, VEGF, *NEGR1*
8	Epigenome & gene–environment interplay (Section 3.9)	DNA-methylation, histone marks and other epigenetic mechanisms—especially after early-life stress (ELS)	*FKBP5*, BDNF exon IV, polygenic-risk × pollution models
9	Translational and Therapeutic Horizons (Section 3.10)	Not a molecular pathway per se, but a translational roundup of drugs and gene-editing tools that act on the above domains	Esketamine, zuranolone, κ-opioid, muscarinic M4 receptor, multi-ancestry GWAS loci
10	Opioidergic Modulation (Section 3.12)	Neuropeptide-based opioid signaling axis modulating stress-, reward-, and affect-related neural circuits	MOR, KOR, DOR

**Table 2:** Monoaminergic Gene–Receptor Findings.

Gene/Receptor	Variant/Strategy	Key Finding	References
*HTR1A*	rs6295 C→G	5-HT1A postsynaptic: inhibitory; predicts SSRI response	[48,49,50]
*HTR2A*	rs6311/rs6313	Insomnia	[51,52,53]
*SLC6A4*	Promoter CpG hyper-methylation	Blunted SSRI response	[50,54]
DRD2/ANKK1	Taq1A (rs1800497)	Anhedonia; ↓ striatal DRD2	[55]
DRD3	Ser9Gly	CC genotype → impaired performance on DRS-2 (Initiation/Perseveration and Construction)	[56]
COMT	Val158Met	Met allele ↑ stress sensitivity	[57]
*SLC6A4* (5-HTTLPR)	S/L promoter VNTR	S allele × stress → ↑ depression risk	[58,59]
TAAR1	Ulotaront/SEP-363856, other TAAR1 agonists	Modulates dopamine, serotonin, glutamate|Antipsychotic, anxiolytic, antidepressant; improves positive, negative, cognitive symptoms	[43,44,45,46,47,60]

**Table 3:** Glutamatergic findings.

Target/ Modulator	Main Findings	Plasticity Interpretation	Clinical Implications in MDD/TRD	References
*GRIN2B*/GluN2B (gene → subunit)	Post-mortem anterior-cingulate samples from MDD show increased GRIN2B mRNA (+32–40%) and a lower *GRIN2A:GRIN2B* ratio; animal work with the C456Y knock-in and other *GRIN2B* variants demonstrates impaired LTD and stress-sensitive hyperconnectivity.	Excess GluN2B skews NMDAR calcium entry toward a “high-gain” state that lowers the threshold for maladaptive metaplasticity.	Identifies extrasynaptic GluN2B as a prime target for rapid-acting or circuit-sparing antagonists.	[86,94]
GluN2B-selective antagonists (e.g., traxoprodil/CP-101,606)	A placebo-controlled human study and parallel chronic-stress mouse models show that a single traxoprodil dose produces ≥1-week symptom relief and durable spine restoration.	Selective dampening of extrasynaptic GluN2B channels permits synaptic NMDAR signalling to drive BDNF-mTOR plasticity without global blockade.	Proof-of-concept for subunit-selective NMDAR drugs that spare cognition while delivering ketamine-like speed.	[95]
*GRM5*/mGluR5	PET studies report a decrease in cortical mGluR5 binding in unmedicated MDD, inversely correlating with symptom severity; ketamine further suppresses the signal in responders.	Reduced mGluR5 tone may be a homeostatic response that lowers IP_3_-Ca^2+^ bursts, resetting metaplastic set-points after ketamine.	Supports trials of mGluR5 negative allosteric modulators (MTEP, GRN-529) as stand-alone or post-ketamine maintenance agents.	[89]
mGluR2/3 antagonists (presynaptic disinhibitors)	Reviews and pre-clinical packets show LY341495, MGS0039 and related inhibitors reproduce ketamine-like behavioural rescue, spine growth and mTOR-BDNF activation without dissociation.	Blocking the autoreceptor “brake” transiently amplifies glutamate bursts that recruit AMPAR and GluN2B-dependent plasticity.	Under exploration as oral, non-dissociative fast-acting antidepressants; may synergise with psychotherapy by opening a plasticity window.	[91,96]
Broad & site-specific NMDAR modulators (ketamine, esketamine, rapastinel, etc.)	Ketamine/esketamine: rapid symptom drop within 2 h, linked to interneuron NMDAR blockade → cortical disinhibition → BDNF-mTOR spine formation; S-ketamine up-regulates BDNF, PSD-95, AKT/mTOR in hippocampus. Rapastinel (GLYX-13) and next-gen analogues act as glycine-site PAMs, delivering 2 h–7 day relief in POC trials without psychotomimetic effects.	All converge on transiently boosting synaptogenesis (“synaptogenic burst”) that normalises circuit throughput.	Intranasal esketamine is FDA-approved for TRD; IV/SC ketamine is widely used off-label. Rapastinel failed phase III but drives development of safer NMDA-site modulators.	[97,98,99]

**Table 4:** GABAergic Findings—loss-of-function lesions in GABA signaling, whether through reduced gene expression or a trafficking-defective variant and pharmacological strategies that restore or fine-tune that same inhibitory tone.

Gene/Receptor	Variant/Strategy	Key Finding	Reference
*GAD1* (GAD67)	Global knock-down (mouse)	Reduced GAD67 lowers cortical GABA and produces depressive-like behaviour	[117]
SST interneuron	Decreased SST mRNA (human sgACC)	Loss of dendrite-targeting inhibition linked to MDD	[118]
MDGA1/*Nlgn2*	Stress-driven MDGA1 over-expression; *Nlgn2* variant	MDGA1 blocks *Nlgn2*, prunes GABA synapses in LHb; reversing interaction rescues depression	[114]
GABA_A receptor–gephyrin	IL-10 deficiency impairs PI3K-AKT-gephyrin trafficking	IL-10 over-expression restores receptor surface delivery and mood	[115]
GABA_A δ subunit	Neurosteroid PAM (zuranolone)	Oral zuranolone yields rapid antidepressant response in adults with MDD	[116]

**Table 5:** Neuropeptide Receptor Findings.

Gene/Receptor	Variant/Strategy	Key Finding (Last 10 Years)	Reference
CRHR1	rs110402 A allele (intronic; enhancer region)	Among healthy young adults, A-carriers show greater amygdala BOLD reactivity to threat faces than GG homozygotes; the effect is amplified by high early-life stress, linking the SNP to limbic hyper-responsivity and mood-disorder risk.	[119]
CRHR1	Early-life stress × rs110402 (multilocus HPA-score)	In a cohort of 308 subjects, a high HPA genetic-risk score that includes rs110402 potentiated the positive correlation between childhood adversity and right-amygdala reactivity, supporting a gene-by-environment pathway to anxiety symptoms.	[120]
CRHR1/CRF1	Pharmacologic—pexacerfont (oral CRF1 antagonist)	A 6-week randomised, placebo-controlled trial in 242 patients with generalised anxiety disorder found no symptomatic advantage for pexacerfont despite good safety and pharmacodynamic target engagement.	[121]
CRHR1/CRF1	Pharmacologic—verucerfont (CRF1 antagonist)	In 39 alcohol-dependent women, verucerfont robustly suppressed cortisol responses but failed to reduce stress-induced craving, illustrating target engagement without clinical efficacy and hinting that biomarker-guided subgroups will be needed.	[122]
CRHR1	DNA-methylation change during CRF1-blockade	In PTSD patients treated with the CRF1 antagonist GSK561679, lower baseline CRHR1 methylation and treatment-related demethylation tracked greater symptom reduction, suggesting epigenetic status may stratify responders.	[123]
*NPY1R*	Elevated amygdala *NPY1R* mRNA (gene-expression endophenotype)	In rhesus macaques, higher *NPY1R* expression in the central amygdala predicted lower anxious temperament and reduced metabolic activity in threat circuits, linking the Y1 receptor to resilience.	[124]

**Table 6:** Hormonal and metabolic receptors that have shown reproducible links to MDD.

Receptor/Pathway	Mechanistic Change in MDD	Key Recent Evidence	Clinical/ Therapeutic Note	Reference
Glucocorticoid receptor (*NR3C1*)	Childhood maltreatment leaves an epigenetic “scar”: higher DNA-methylation in the *NR3C1*-1F promoter lowers GR expression and perpetuates cortisol hyper-secretion	2-year, 3-wave study (n = 370 adolescents) shows *NR3C1* methylation mediates the maltreatment → depression link	Highlights GR resistance as a treatable node (e.g., psychotherapy, mifepristone trials)	[126]
Estrogen receptors (ER-α/β)	Perimenopausal estradiol fall disrupts ER-dependent synaptic plasticity in fronto-limbic circuits	Multidisciplinary guideline summarises RCT data showing transdermal estradiol relieves perimenopausal depressive symptoms	Defines a “hormone-window” where add-on estradiol can augment standard antidepressants	[136]
Thyroid-hormone receptors (TR-α/β)	Low-grade “cerebral hypothyroidism” slows cortical metabolism	Network meta-analysis of 65 RCTs finds liothyronine (T3) among the most effective augmentation agents for treatment-resistant depression	Supports low-dose T3 (25–50 µg/day) augmentation in refractory MDD	[137]
Central insulin receptor signalling	Insulin resistance (IR) associates with BBB leakage and worse mood	MRI study links extensive BBB leakage + IR to more severe bipolar depression	Points to metformin/lifestyle or GLP-1RA use to normalise IR and mood	[138]
GLP-1 receptor	Gut–brain incretin signalling modulates neuroinflammation and reward circuits	Meta-analysis of 6 trials (n ≈ 2070) shows GLP-1R agonists reduce depression scores vs. controls (SMD = −0.12)	Liraglutide/semaglutide offer weight-independent mood benefits; trials in primary MDD underway	[133]
PPAR-γ receptor	Nuclear receptor that couples metabolism and inflammation	Double-blind RCT (n = 56) found pioglitazone safe but not superior to placebo for bipolar depression; leptin change tracked symptom change	Suggests PPAR-γ modulation may help selected metabolic-inflammatory subgroups	[134]
Melatonin MT1/MT2 (plus 5-HT_2_C antagonism)	Circadian misalignment and metabolic dyscontrol in MDD	Large real-world VIVALDI cohort confirms agomelatine’s antidepressant efficacy and good tolerability	Chronobiotic strategy useful where sleep–wake disruption co-drives depression	[135]

**Table 7:** Immune & Inflammatory Findings—Across innate-immune (TLR4, *P2RX7*/NLRP3), cytokine (IL-6), metabolic (FFAR2, IDO1) and microbiota-directed nodes, convergent evidence indicates that immune activation and gut-derived ligands constitute a modifiable pathway driving depressive pathology. Precision interventions such as anti-cytokine biologics, P2X7/NLRP3 inhibitors, SCFA supplementation and targeted probiotics are now entering clinical testing to rebalance these immuno-inflammatory interfaces.

Gene/ Receptor	Variant/Strategy (Last-Decade Evidence)	Key Finding Relevant to Depression	Reference
IL-6 (IL6)	Persistently elevated baseline serum IL-6 (systemic low-grade inflammation)	High IL-6 and CRP at baseline predicted a new MDD diagnosis nine years later in a population cohort, underscoring cytokine priming as a risk factor	[154]
TLR4 (LPS sensor)	“Leaky-gut” translocation of LPS, zonulin ↑/I-FABP ↑ in adolescents with MDD	Raised gut-permeability markers suggest endotoxin leakage that can activate TLR4 on monocytes and microglia, fuelling neuro-inflammation	[155]
*P2RX7* → NLRP3 inflammasome	ATP-driven P2X7 activation/genetic or drug blockade	Review of 2024 summarises human & rodent data: P2X7-triggered NLRP3 activation elevates IL-1β; selective antagonists or knock-out reverse anhedonia & despair-like behaviour	[147]
FFAR2 (GPR43)	Exogenous short-chain fatty acid (butyrate) supplementation	SCFA administration restores microglial homeostasis, repairs BBB and abolishes depressive-like behaviour in stress- or diet-induced mouse models; human MDD plasma shows SCFA deficit	[145]
IDO1/Kynurenine pathway	Microbiome-driven ↑ Kynurenine: Tryptophan ratio	Multi-omics study links gut dysbiosis to skewed kynurenine metabolism and higher depressive severity, implicating IDO1 activation as a neurotoxic switch	[156]
Microbiota (multi-strain probiotic)	8-week adjunctive probiotic RCT in SSRI partial responders	Randomised trial (n = 49) showed greater HAM-D improvement and reduced plasma IL-6 vs. placebo, supporting “psychobiotic” augmentation	[152]

**Table 8:** Neurotrophic and synaptic gene findings implicated in MDD.

Gene/Receptor	Variant/Strategy (Last-Decade Data)	Key Finding Relevant to Depression	Reference
BDNF	Promoter CpG-87 hyper-methylation (exon IV)	Hypermethylation dampens BDNF transcription; in a 199-patient cohort, it predicted poorer SSRI/SNRI response, whereas demethylation tracked remission.	[172]
*NTRK2* (TrkB)	rs2579372 polymorphism	Risk allele linked to smaller bilateral hippocampal volumes and higher odds of treatment resistance; volume statistically mediated the genotype-outcome link.	[165]
Synaptic scaffolds (PSD95/*DLG4*, *SHANK3*, *GRIA2*)	Cell-type transcriptional shift (snRNA-seq, sgACC)	Single-nucleus TWAS of 320 post-mortem brains showed coordinated down-regulation of excitatory-synapse genes and up-regulation of microglial pruning genes in MDD.	[173]
mTORC1 pathway	Ketamine ± rapamycin manipulation	Ketamine’s rapid spine gain and antidepressant behaviour required mTORC1; intra-PFC rapamycin blocked both molecular and behavioural effects in rodents.	[174]
VEGF	Baseline plasma level and treatment response	Lower baseline VEGF predicted non-response, while responders showed VEGF increases after ECT/rTMS, implicating inducible angiogenic signalling in recovery.	[175]
NEGR1	Brain over-expression	Elevating NEGR1 in ventral hippocampus induced anxiety-/depression-like phenotypes and synaptic dysfunction; knock-down or rescue reversed stress anhedonia.	[171]

**Table 9:** Air-pollution interactions with genetic risk.

Study	Sample (Follow-Up)	Environmental Exposure	Genetic Measure	Key Result
Gao et al., 2023 [182]	398,241 UK Biobank adults (median 8.7 y)	Annual PM_2.5_, NO_x_, NO_2_	Genome-wide PRS for depression/anxiety	Highest pollution quintile plus high PRS: HR 1.11 (95% CI 1.05–1.18) for incident mood-anxiety disorders; significant additive interaction
Fu et al., 2022 [181]	~500,000 adults prospective cohort	PM_2.5_, PM_10_, NO_2_, NO_x_	11-SNP genetic-risk score	Air pollutants + high genetic risk produced relative-excess risk due to interaction (RERI) 0.10–0.15 for depression

**Table 10:** Pipeline receptor-targeted antidepressants.

Gene/Receptor Target	Drug/Strategy	Key Recent Finding	References
NMDA (GluN2B-containing) receptor	Esketamine nasal spray, non-competitive NMDA antagonist	Phase-3 ASPIRE trials showed a clinically meaningful fall in depressive symptoms and suicidal ideation 24 h after the first dose in severely ill MDD patients	[197]
GABAA receptor (extrasynaptic δ-containing)	Zuranolone oral neuro-steroid PAM	A phase-3 RCTs met the MADRS primary end-point after a 14-day, once daily 50 mg course with benefits visible by Day 3	[199]
Kappa-opioid receptor (KOR)	Aticaprant (JNJ-67953964) antagonist	Adjunctive phase-2 trial delivered a −5.6 MADRS advantage over placebo at Week 4; a 2025 phase-3 programme was halted for futility, illustrating target-risk	[200]
Muscarinic M4 receptor	Emraclidine (CVL-231) positive allosteric modulator	Brain-penetrant, highly selective M4 PAM produced antipsychotic-like efficacy without cognitive burden in early trials and is now moving into mood-disorder studies	[201,202]
Multi-ancestry GWAS loci (354 genes)	Genomic discovery platform	2024 mega-GWAS mapped 354 depression-risk loci enriched for synaptic-vesicle and neurodevelopmental genes—a roadmap for future receptor-based drugs	[203]

**Table 11:** Opioid Receptors and Targeted Antidepressant Strategies.

Receptor	Typical State in Depression	Functional Consequence	Therapeutic Logic	References
MOR	Hypofunction/partial engagement	Reduced antidepressant and anxiolytic tone; possible increased suicidal ideation	Restore MOR signaling (e.g., low-dose buprenorphine, partial agonists)	[214]
KOR	Overactivated by stress-induced dynorphins	Dysphoria, anhedonia	Block KOR (e.g., aticaprant)	[200]
DOR	Reduced expression in key brain regions (amygdala, hippocampus)	Impaired mood regulation, increased anxiety, cognitive-emotional deficits	Activate DOR (investigational agonists)	[217]

**Table 12:** Cross-Domain Summary of High-Confidence Gene–Receptor Associations.

Domain	Gene/ Receptor	Robustly Documented Variant/Alteration	Main Phenotypic Signal in Affective Pathology	References
Serotonin	*HTR1A*	rs6295 (C-1019G, promoter)	increase presynaptic 5-HT1A, decrease cortical binding; slower or poorer SSRI response	[20,228]
Dopamine	DRD2/ANKK1	rs1800497 (Taq1A, A1)	5–12% decrease striatal D2/3 BP; higher anhedonia & reward-blunting in MDD	[74,229]
Glutamate	GRIN2A	rs7192557 (intronic)	Replicated in tardive dyskinesia; only nominal signals for MDD—not GW-significant	[230]
GABA	GABRA5	α5-GABA_A NAMs	Rapid antidepressants in animals, restoring synaptic strength much like ketamine, yet without NMDAR blockade	[231]
Neuropeptide/Stress	CRHR1	rs110402	A-allele buffers, G-allele amplifies childhood-abuse risk for adult MDD (G × E)	[232,233]
Hormonal (HPA axis)	*NR3C1*	Hyper-methylation of promoter exon 1F in neonates of depressed mothers	Alters infant cortisol reactivity; candidate mechanism for perinatal MDD transmission	[196]
Innate immune	TLR4	Asp299Gly (D299G)	Loss-of-function variant reduces pro-inflammatory signaling; mixed evidence for mood effects—no melancholic specificity, possibly associated with inflammation-mediated depression	[234]
Neurotrophic	BDNF	Val66Met (rs6265)	Met carriers show attenuated rapid antidepressant & anti-suicidal response to ketamine	[235,236]

## Data Availability

No new data were created or analyzed in this study. Data sharing is not applicable to this article.

## Abbreviations

MDD	Major Depressive Disorder
5-HT	5-Hydroxytryptamine (Serotonin)
BDNF	Brain-Derived Neurotrophic Factor
COMT	Catechol-O-Methyltransferase
CRHR1	Corticotropin-Releasing Hormone Receptor 1
DRD2/3	Dopamine Receptor D2/D3
ER α/β	Estrogen Receptor α/β
*FKBP5*	FK506 Binding Protein 5
*GAD1*	Glutamic Acid Decarboxylase 1 (GAD67)
*GRIA2*	Glutamate Ionotropic Receptor AMPA Type Subunit 2
*GRIN2B*	Gene located on the short arm (called “p”) of the 12th chromosome at 12p13.1
*GRM5*	Metabotropic Glutamate Receptor 5
IDO1	Indoleamine 2,3-Dioxygenase 1
IL 6	Interleukin 6
MDGA1	MAM Domain-Containing Glycosylphosphatidylinositol Anchor 1
*Nlgn2*	Neuroligin 2
NEGR1	Neuronal Growth Regulator 1
*NPY1R*	Neuropeptide Y Receptor 1
*NR3C1*	Nuclear Receptor Subfamily 3 Group C Member 1
*NTRK2* (TrkB)	Neurotrophic Receptor Tyrosine Kinase 2
PPAR γ	Peroxisome Proliferator-Activated Receptor Gamma
PSD95/*DLG4*	Postsynaptic Density Protein 95/Discs Large Homolog 4
*SLC6A4*	Solute Carrier Family 6 Member 4 (Serotonin Transporter)
SST	Somatostatin Interneuron
TAAR1	Trace Amine Associated Receptor 1
TLR4	Toll-Like Receptor 4
TR α/β	Thyroid Hormone Receptor α/β
VEGF	Vascular Endothelial Growth Factor
GLP 1 receptor	Glucagon-Like Peptide 1 Receptor
MT1/MT2	Melatonin Receptors 1 and 2
5 HTTLPR	5-Hydroxytryptamine Transporter-Linked Polymorphic Region
VNTR	Variable Number Tandem Repeat
DRS 2	Dementia Rating Scale 2
ANKK1	Ankyrin Repeat and Kinase Domain Containing 1
GluN2B	Glutamate receptor, ionotropic, NMDA 2B
GRIN2A	Glutamate Ionotropic NMDA Receptor Subunit 2A
LTD	Long-Term Depression
NMDAR	N-Methyl-D-Aspartate Receptor
mTOR	Mechanistic Target of Rapamycin
PET	Positron Emission Tomography
IP_3_	Inositol Triphosphate
Ca^2+^	Calcium ion
AMPAR	α-Amino-3-hydroxy-5-methyl-4-isoxazolepropionic acid Receptor
PAM	Positive Allosteric Modulator
POC	Proof of Concept
IV/SC	Intravenous/Subcutaneous
TRD	Treatment-Resistant Depression
sgACC	Subgenual Anterior Cingulate Cortex
LHb	Lateral Habenula
GABA_A	Gamma-Aminobutyric Acid type A receptor
PI3K	Phosphoinositide 3-Kinase
AKT	Protein Kinase B
5-HT_2_C	Serotonin receptor 2C
BBB	Blood–Brain Barrier
IR	Insulin Resistance
T3	Triiodothyronine
CRP	C-Reactive Protein
LPS	Lipopolysaccharide
I-FABP	Intestinal Fatty Acid Binding Protein
*P2RX7*/P2X7	P2X7 Purinergic Receptor
NLRP3	NLR Family Pyrin Domain Containing 3
IL 1β	Interleukin 1 beta
FFAR2 (GPR43)	Free Fatty Acid Receptor 2/G Protein-Coupled Receptor 43
SCFA	Short-Chain Fatty Acid
HAM-D	Hamilton Depression Rating Scale
*SHANK3*	SH3 and Multiple Ankyrin Repeat Domains 3
snRNA-seq	Single-Nucleus RNA Sequencing
TWAS	Transcriptome-Wide Association Study
mTORC1	Mechanistic Target of Rapamycin Complex 1
PFC	Prefrontal Cortex
ECT	Electroconvulsive Therapy
rTMS	Repetitive Transcranial Magnetic Stimulation
PRS	Polygenic Risk Score
PM_2.5_/PM_10_	Particulate Matter ≤2.5 µm/≤10 µm
NO_2_/NO_x_	Nitrogen Dioxide/Nitrogen Oxides
HR	Hazard Ratio
CI	Confidence Interval
RERI	Relative Excess Risk due to Interaction
KOR	Kappa-Opioid Receptor
RCT	Randomized Controlled Trial
MADRS	Montgomery–Åsberg Depression Rating Scale
GWAS	Genome-Wide Association Study
MOR	Mu-Opioid Receptor
DOR	Delta-Opioid Receptor
NAMs	Negative Allosteric Modulators
BP	Binding Potential
G × E	Gene-by-Environment interaction
